# Re-discussion of servitization strategy and firm performance

**DOI:** 10.3389/fpsyg.2022.1022648

**Published:** 2022-10-13

**Authors:** Kang Li, Jinfeng Wang, Lijie Feng, Lei Zhu

**Affiliations:** ^1^Institute of Logistics Science and Engineering, Shanghai Maritime University, Shanghai, China; ^2^China Institute of FTZ Supply Chain, Shanghai Maritime University, Shanghai, China; ^3^School of Economics and Management, Shanghai Maritime University, Shanghai, China

**Keywords:** servitization strategy, manufacturing firm performance, servitization paradox, moderator variable, meta-analysis

## Abstract

Servitization innovation is critical for manufacturing firms to strengthen their sustainable competitive advantage in a dynamic business environment. Current research on the relationship between servitization and firm performance has matured, but many conclusion remain divergent. That cannot only hinder the development of servitization theory, but also make manufacturers lack a scientific basis for deciding whether to develop servitization. Thus, this study aims to systematically analyze the quantitative research results in this field through Meta-analysis methods to reveal the reasons for the disagreement. After collecting 59 independent articles on servitization and firm performance, this study performed statistical analysis using Meta-analysis. Then, the relationship between servitization and firm performance was explored, as well as the effects of different potential moderating variables. The moderate positive relationship between servitization strategies and their different orientations and firm performance is found. For the moderating variables, the servitization strategy has a more significant effect on non-financial performance. And they are more correlated when there are mediator variables. The impact of firm servitization transformation in developing regions is better than in developed areas. A stable market environment is more beneficial to the servitization transformation. The transformation effect of high-tech manufacturing is better than that of traditional manufacturing. And the transformation effect of large companies is better than that of small and medium-sized companies.

## Introduction

In the Industry 4.0 era, the servitization of manufacturing is a new business model and production pattern (Weking et al., 2020). In this context, more manufacturers are considering its servitization with a strategic change from manufacturing products simply to providing product service (Nájera-Sánchez et al., 2020; Liu et al., 2022). Because the homogenization of tangible products is an inevitable trend, it is only to create a competitive advantage of differentiation among them through service innovation (Baines et al., 2009). In other words, firms implement a servitization strategy to provide integrated solutions for customers and create new value (Zhang et al., 2021). This strategy has become essential for the manufacturing industry to achieve industrial upgrading and maintain sustainable development (Hu et al., 2022). Additionally, for traditional manufacturing firms, a servitization strategy can help them break through the dilemma of low-cost and homogeneous product competition. It helps to compensate for their loss of competitive advantage in the value chain, thereby obtaining new opportunities for profit growth (Vendrell-Herrero et al., 2017). Therefore, many countries have formulated corresponding policies to actively promote the servitization process of their own manufacturing industries in order to gain advantages in international competition, such as Germany’s “Industry 4.0” strategy and China’s “Made in China 2025.” Meanwhile, the servitization strategy has attracted a lot of attention from scholars.

However, there are different conclusion about the impact of servitization strategy on firm performance (Feng et al., 2020; Huang et al., 2020). Numerous empirical studies support that implementing a firm’s servitization strategy positively impacts its performance (Raddats et al., 2019; Wang et al., 2022). But some literature shows that there is no positive correlation between them. For example, Benedettini et al. (2017) suggested that servitization is challenging to generate the expected benefits and negatively impacts firms. Some scholars have explored the relationship between servitization and firm performance based on different types of servitization, with different conclusion. For example, Ting (2019) concluded that both product-oriented and customer-oriented servitization strategies could improve firm performance. Sousa and da Silveira (2017) have found that a product-oriented servitization strategy has no significant effect on firm performance. In addition, due to the existence of various moderating variables that remain unidentified, the relationship between servitization and firm performance is inconclusive (Huang et al., 2020). It has prompted scholars debate whether the relationship between servitization strategies and firm performance is linear, and their conclusion are inconsistent (Feng et al., 2021).

The above analysis shows that the impact mechanism of a firm implementing a servitization strategy on firm performance is unclear. It hinders the development of servitization theory and makes manufacturers lack a scientific decision-making basis for whether to develop servitization. Therefore, it is worth systematically analyzing their relationship and underlying mechanisms. Moreover, the servitization strategy has now become an important tool for manufacturing firms to cope with the challenges caused by economic globalization, individualized customer demands, and the rapid development of emerging technologies in the context of Industry 4.0 (Li et al., 2020; Grabowska and Saniuk, 2022). However, whether servitization and its different oriented servitization strategies can improve firm performance remains unknown. Furthermore, whether different potential variables impact the relationship between servitization and firm performance remains to be investigated. Therefore, this study attempts to systematically analyze their relationship based on a meta-analysis of the empirical literature and to answer the following research questions: Is the relationship between servitization strategy and firm performance influenced by different strategic orientations and potential variables, and how is it affected?

The structure of this study is organized as follows. After the introduction, this study presents the literature review of servitization and the influencing factors in Section “Literature review,” and proposes the research hypothesis and theoretical model. The research design and data processing process is described in Section “Research design,” and then the results of this study are shown in Section “Results.” The discussion of this study is in Section “Discussion,” and the conclusion and limitations are presented in Section “Conclusion and limitation.”

## Literature review

To support the hypothesized relationships and models we have proposed, this section presents a brief literature review on servitization and its different oriented strategies and influencing factors.

### Servitization

Servitization was proposed by Vandermerwe and Rada (1988), which refers to the change in the role of manufacturing firms from product manufacturers to product service providers. It provides operation and maintenance services around the entire life cycle of its core products (Salonen et al., 2017). And it promoted the transformation and upgrading of manufacturing firms through service innovation. Relevant theoretical studies on servitization mainly focus on the connotation, driving factors, and future research directions. For example, in terms of the connotation of servitization, Wang et al. (2018) defined servitization as the process of transforming business strategies with service-oriented logic. Chou (2021) regarded servitization as a service-oriented business model innovation. In terms of driving factors, Baines et al. (2020) investigated the process of organizational change generated by servitization to analyze the drivers of servitization. Nájera-Sánchez et al. (2020) identified value co-creation as an important driving factor for firms to develop servitization *via* a bibliographic coupling analysis. In terms of future research directions, Khanra et al. (2021) recommended actionable future research agendas on servitization by systematically analyzing the servitization literature. Zhang et al. (2021) explored the future research agenda of servitization through a literature review and concluded that topics such as actor engagement and digital servitization are the future research directions.

Currently, the rise of the service economy has led scholars to focus on the practical application of servitization (Feng et al., 2020). Many scholars have confirmed that servitization can bring significant competitive advantages to firms. Rosa et al. (2019) reported that servitization helps manufacturing firms break current competitive landscapes, which can satisfy customer demands while enhancing firm value. Bustinza et al. (2015) found that servitization offers firms the opportunity to generate a sustainable competitive advantage based on an international survey of manufacturing practices. The success of service innovations by companies such as IBM: Armonk, New York, NY USA; Hewlett-packard: Palo Alto, CA, USA; Michelin: Clermont-Ferrand, France supports this view (Wang et al., 2022). In addition, Gomes et al. (2019) found that implementing territorial servitization strategies by medium-sized firms (SMEs) is conducive to developing economies of scale, enabling them to compete with larger manufacturing firms. Thus, we hypothesize that:

Hypothesis 1. Servitization strategy positively impacts on the firm performance.

### Strategic orientation of servitization

With the development of servitization theory, scholars have concluded that servitization strategies are not of a single type and their impact on firm performance is different. Mathieu (2001) classified servitization strategies into product-oriented and customer-oriented according to the service objectives of manufacturers. This division criterion received widespread support from scholars. Although other scholars subdivided servitization strategy types from different perspectives later, no significant differences were found with the classification of Mathieu (2001). Therefore, this study classified servitization types into two categories: product-oriented servitization strategies and customer-oriented servitization strategies.

#### Product-oriented servitization

Product-oriented servitization strategy emphasizes product as the focus of service. The key is to ensure the normal use of products by providing the underlying services that support the supplier’s product. This strategy also aims to reinforce product functionality and differentiation for manufacturers, with typical characteristics such as standardization, and low complexity. Typical examples are after-sales services such as product installation, testing, and maintenance. There are studies have shown that product-oriented servitization strategies do not affect a firm financial performance (Sousa and da Silveira, 2017). However, more studies indicated that firms could reduce product failure risk and customer utilization cost by providing product-oriented services (Juhong et al., 2017). It can help optimize the operational efficiency of client products, improve the core value of products, and enable firms to succeed in the competition. Besides, Cusumano et al. (2015) reported that underlying services provided by firms could improve the satisfaction and loyalty of their customers, which achieves a steady increase in turnover. Therefore, we hypothesize that:

Hypothesis 2a. Product-oriented servitization strategy positively impacts the firm performance.

#### Customer-oriented servitization

Customer-oriented servitization strategy emphasizes customers as the center of service. This strategy helps customers achieve their goals by providing advanced services supporting their actions. Customization, high complexity, and high relationship intensity are typical characteristics of this strategy. Such as information consulting, process optimization, and other services. Although customer-oriented advanced services seem to be a massive challenge for firms with their resources and capabilities, they will also bring higher benefits to them. Sjödin et al. (2016) indicated that services offered by supporting customer activities help firms to identify potential marketing opportunities along the customer activity chain. This will allow firms to create a competitive advantage that is hard to beat. Similarly, Yongyi et al. (2016) found that customer-oriented service innovation can build closer customer-firm relationships and promote value reorganization of products and services, which helps firms gain more economic benefits. Therefore, we hypothesize that:

Hypothesis 2b. Customer-oriented servitization strategy positively impacts the firm performance.

### Influencing factors

Due to the limitations of samples, sampling time, and other factors, it is not easy to comprehensively find the moderating factors that affect servitization strategy and firm performance (Feng et al., 2020). This may be an important reason for the divergent conclusion of the available literature. Based on this, this study integrates various factors that influence the relationship between servitization firm performance and explores their moderating effects through Meta-analysis. These influences are measurement factors, mediating variables, sampling area, market environment, technical level, and firm scale.

#### Measuring factors

As a visual indicator to measure the effectiveness of servitization strategy, firm performance mainly includes financial and non-financial performance. Among them, financial performance mainly includes profitability, return on investment and assets, etc. The main aspects of non-financial performance are market share, product innovation, and customer satisfaction. Servitization strategies optimize the quality of a company’s products, which in turn improves customer satisfaction and thus has a direct impact on its non-financial performance (Hallencreutz and Parmler, 2021). However, due to the servitization paradox, financial performance improvement often lags behind non-financial performance (Kastalli and Van Looy, 2013). Lin et al. (2018) found that servitization helps manufacturing firms to improve their operational performance, but profitability is less affected. Therefore, it can be considered that servitization has a more significant effect on non-financial performance than financial performance, which is influenced by the indicators that measure firm performance. Accordingly, we hypothesize that:

Hypothesis 3. The servitization strategy-firm performance relationship is influenced by the type of performance measurement. The impact of servitization strategy on the firm performance is more significant when measured by non-financial indicators.

#### Mediating variables

Although many studies concluded that the implemented servitization strategies benefit firm performance, there is no consensus on the degree of correlation between them. Thus, some scholars have begun to consider whether there are other mediating variables that can change the effect of servitization strategies on firm performance. Juhong et al. (2020) concluded that business model innovation significantly affects the relationship between servitization strategy and firm performance. Nan et al. (2016) empirically studied the mediating role of internal and external knowledge co-creation between service provision and service innovation performance. It can be found that current scholars are primarily interested in investigating the effect of the presence of mediating variables on the relationship between servitization and firm performance. And the effect is more significant when the mediating variables are present. Therefore, we propose the following hypothesis:

Hypothesis 4. The servitization strategy–firm strategy performance relationship is influenced by the existence of mediating variables. The impact of servitization strategy on the firm performance is more significant when there are mediating variables.

#### Sampling area

The concept of manufacturing service originated in developed countries and gradually went global with the rise of the service economy. However, with different economic development levels of countries or regions, the implementation effects of servitization strategies by manufacturing firms are also different (Hong et al., 2014). Most manufacturing firms in developed areas, represented by Europe and the United States, have entered the era of service economy. Manufacturing firms in developing areas are still in the exploratory stage of servitization due to their weak industrial base. However, since developing areas are emerging economies with huge development potential, there are more potential development opportunities. Meanwhile, emerging developing countries or regions represented by China pay much attention to solving difficulties for the servitization transformation of manufacturing firms. These actions provide new power for the high-quality development of service-oriented firms. It also implies that the servitization transformation of manufacturing industries in developing countries has a more considerable profit margin. Consequently, we hypothesize that:

Hypothesis 5. The servitization strategy-firm performance relationship is influenced by the economic conditions of the sampled regions. The servitization of manufacturing firms in developing regions has a more significant impact on their performance.

#### Market environment

The market environment directly affects the implementation effect of the servitization strategy of manufacturing firms. Servitization transformation is a reliable way for manufacturing firms to gain a competitive advantage in a stable market environment (Zhang et al., 2019). In a stable market environment, it is not easy to gain a significant competitive advantage for manufacturing firms by focusing only on a product-focused cost leadership strategy (Smith, 2013). But servitization can help manufacturers satisfy customers’ customized demands while enhancing the added value of products and customer satisfaction (Buganza et al., 2020). Instead, firms often adopt conservative strategies for growth, given the many uncertainties in a volatile market environment, aiming to reduce business risk (Wang et al., 2018). Since the financial crisis in 2008, economic development worldwide has been constrained to vary degrees. It has exacerbated market volatility, making the risks of manufacturing servitization transformation even more unpredictable. Therefore, this study sets the period before 2008 as the stable period and the period after 2008 as the turbulent period. Thus, we propose the following hypothesis:

Hypothesis 6. The servitization strategy-firm performance relationship is influenced by the market environment. The servitization of manufacturing firms in a stable period market environment has a more significant impact on their performance.

#### Technical level

The effectiveness of servitization for manufacturing firms will differ depending on its technical level (Frank et al., 2019). Restricted by the technical level, traditional manufacturing firms’ products are slowly changing and severely homogenized. The simple services it provides can be easily imitated by competitors, making it difficult to achieve desired results. In contrast, high-tech manufacturing firms are knowledge-intensive, technology-intensive, fast product iteration, and have high added value. This gives it a higher marginal substitution effect in the servitization process, which can bring higher performance to firms (Chen and Zhang, 2021). And it provides O&M services that often have high technical barriers which are difficult to imitate by other firms. The synergies created by knowledge and technology spillovers may also help firms reduce their service costs. In addition, high-tech manufacturing firms, their customers often need more service support, which also provides a potential demand market for its servitization transformation (Li et al., 2019). Accordingly, we hypothesize that:

Hypothesis 7. The servitization strategy-firm performance relationship is influenced by the technical level. The servitization of high-tech manufacturing firms has a more significant impact on their performance.

#### Firm scale

Firm scale is an important factor affecting the implementation effect of servitization strategy. In general, SMEs are often in a passive position in servitization. Even if it motivates to development of servitization, it is difficult to achieve the expected results given the constraints of multiple factors such as resources, capacity, and market (Michalik et al., 2019). In contrast, large manufacturing firms have a wide variety and number of products with promising service markets related to them. Also, abundant resources and strength can guarantee firms resist risks when facing many challenges of the servitization process (Jovanovic and Morschett, 2022). Therefore, we hypothesize that:

Hypothesis 8. The servitization strategy-firm performance relationship is influenced by the firm scale. The servitization of large firms has a more significant impact on performance.

### Theoretical model

**FIGURE 1:**
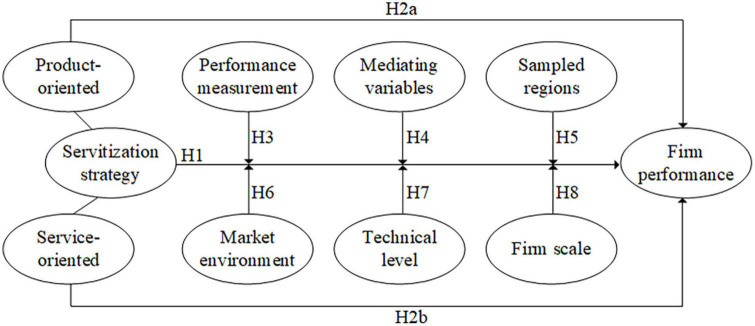
Theoretical model of influencing factors.

Based on the comprehensive analysis of previous sections and research hypotheses, a theoretical model was established, as shown in Figure 1. Servitization strategy was an independent variable, including product-oriented servitization strategy and customer-oriented servitization strategy, while firm performance was a dependent variable. And the influencing factors were moderating variables, including measurement factors, mediate variables and sampling area, market environment, technical level, and firm scale.

## Research design

This section introduces the Meta-analysis method used in this study and then describes the research data’s sampling, screening, coding, and processing process.

### Research methods

The merge statistic is the crucial idea of the Meta-analysis method (Feng et al., 2020). Using statistical concepts and methods to analyze different empirical results of a specific research topic empirically is the essence of this method. It allows the relationship between different empirical study variables to be clarified further and makes up for the shortcomings of traditional review studies. This method has been widely used in education, psychology, and evidence-based medicine. The typical process of meta-analysis is:

1. Research question identification. Research questions should be discovered from more controversial areas of research. Scholars then make predictions about possible relationships between variables and propose reasonable hypotheses.
2. Data Preparation. The empirical literature related to the research questions is first collected systematically and comprehensively from literature databases. After that, the retrieved literature is screened to control the sample quality, and the screened literature is coded.
3. Data processing. This step consists of extracting and converting statistics that characterize the relationship between the explanatory and the explained variables. Then the confidence correction is applied to each effect value.
4. Overall and moderating effects tests. This step starts with the publication bias test and heterogeneity test. Then, the overall and moderating effects are tested based on the test results to verify the proposed research hypotheses.
5. Analysis and discussion of results. This step is to analyze the results and give a reasonable interpretation.

In this study, the empirical studies’ findings on the servitization strategy-firm performance relationship are inconsistent. And there are differences between the conclusion in the strength of the relationship and even in the research direction. The potential moderating variables and their roles are also unclear. Besides, limited studies incorporate Chinese literature into the Meta-analysis of the servitization strategy-firm performance relationship. Therefore, this study applies the Meta-analysis method to synthesize relevant empirical studies, including Chinese literature and English literature. The relationships between them and the effects of potential moderating variables can be further explored comprehensively. It helps to provide valuable insights for service innovation for Chinese manufacturing firms. Comprehensive Meta Analysis 2.0 (CMA 2.0) software was used for the data processing work in this study.

### Sampling

In this study, the literature data used for Meta-analysis were obtained from mainstream English and Chinese literature databases, respectively. The complementarity of English and Chinese literature data helps to search for more complete and comprehensive research literature data as much as possible. Here, we have used purposive sampling techniques based on the manual retrieval of literature to obtain literature data on servitization and firm performance in this study. The specific literature search steps are as follows. First, we retrieved relevant English articles from Web of Science, Elsevier, and other databases with search terms related to servitization and performance. For example, “Servitization,” “Service orientation,” “Service transformation,” etc. Then, we take the China National Knowledge Infrastructure (CNKI) as the data source for collecting Chinese literature data and use different search queries related to servitization and performance to obtain relevant Chinese articles. In the process of retrieving Chinese literature, economic, and management science was selected as the category of literature classification, and the journal grade was limited to core journals or above. Among them, there is no starting point for the retrieval time, and the deadline is 2021. In addition, we reviewed the review articles and references of the acquired articles related to the servitization strategy field. And we compared the acquired literature with them, aiming to avoid the omission of the important literature. Overall, we obtained 241 initial articles, including 137 English and 104 Chinese articles.

To improve the precision of the sample, according to the research of Pigott and Polanin (2020) on how to perform a meta-analysis, this study has been further filtered according to the following criteria: (1) The study must be an empirical study. Because an empirical article with standardized research processes can provide key sample size and correlation coefficients, or other statistics that can be transformed, such as regression coefficients and path coefficients (Field and Gillett, 2010). (2) Contents of the collected articles should be relevant to the servitization strategy-firm performance relationship studies. In addition, articles with servitization as a mediator or moderator and withdrawn articles should be excluded. (3) The samples are independent of each other. If multiple articles use the same sample, the journal with the higher impact factor will be selected. If there are two or more independent samples in a literature, the study samples related to this article will be selected for analysis. A total of 59 articles (including 31 English articles and 28 Chinese articles), 108 effect sizes and 30,278 independent samples were finally screened.

### Coding and processing

#### Coding

This study was coded for the sample literature by two scholars familiar with Meta-analysis to minimize the error in the subsequent analysis according to the coding rules of Wang et al. (2018) and Feng et al. (2020). Coding content includes qualitative information and quantitative information. Qualitative information involves items describing publication information (such as title, author, date, and publication), items describing sample characteristics (such as performance measure type, country and region, date of data collection, industry, and firm size), and items describing study content (such as variable, model, and conclusion). Quantitative information involved sample size, variable reliability, correlation coefficients, and other effect values that could be translated into correlation coefficients (such as regression coefficients, path coefficients, etc.). Then, the coding results were cross-compared by two scholars in the field beside the coders, and the consensus reached 91.52%. Our coding consensus rate is above the 90% agreement rate suggested by Seibert et al. (2011). In other words, the higher the consensus among the different coding results, the more reliable the data coding accuracy is proven to be (O’Connor and Joffe, 2020). It shows that the research can avoid the influence of the subjective cognitive differences of the coders on the analysis results.

#### Data processing

After obtaining the coded data of the sample literature, it is necessary to identify the statistics that can characterize the relationship between variables. Because the study indicators differed, a uniform effect size, the correlation coefficient, was required. After unifying the converted effect values, the correlation coefficients obtained in each empirical literature also need to be corrected for reliability. This operation aims to reduce the attenuation bias caused by scale reliability defects.

In addition, for some literature that did not provide variable reliability, this study used the weighted reliability of other research samples of the same scale instead. Meanwhile, the literature with the following conditions was excluded: Literature that did not provide variable reliability and failed to find sample-weighted reliability of other studies for the same scale. Literature whose regression coefficients are not in the (−0.5, 0.5) interval. Some literature has an absolute value of the transformed effect size greater than 1.

**FIGURE 2:**
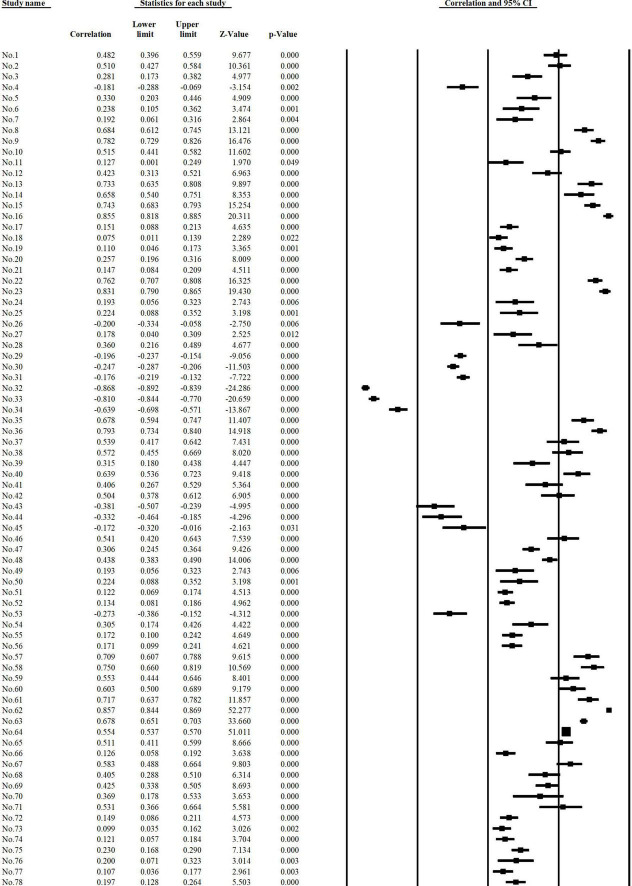
Partial forest map of sample literature.

Moreover, to improve the accuracy of the conclusion, literature with effect values that lie in the 95% confidence interval but significantly cross the zero cut-offs (outliers) needs to be excluded. Finally, the number of selected sample literature was 59 (including 31 in English and 28 in Chinese), the effect size was 108, and the number of independent samples was 30,278. And the forest plot of the sample literature part of the meta-analysis is shown in Figure 2.

## Results

In this section, publication bias and heterogeneity tests were first performed to test the effects of the sample literature. Then, to test the hypothesized relationships and models proposed in this study, this section conducts the overall effects and moderating effects tests based on publication bias and heterogeneity tests, respectively.

### Publication bias

**FIGURE 3:**
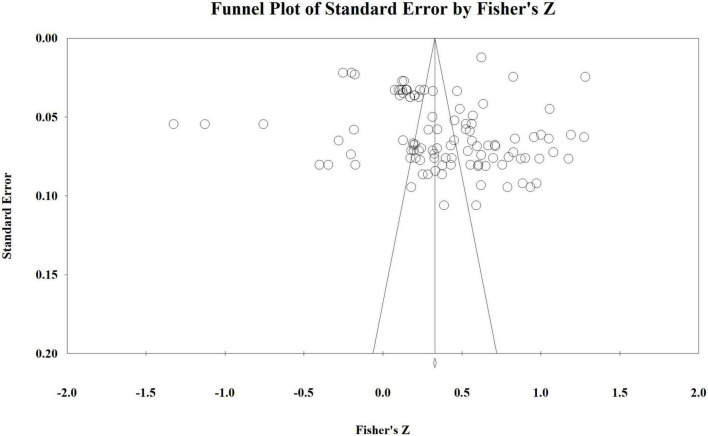
Funnel plot.

Publication bias is one of the most common systematic errors in a meta-analysis, which makes it impossible for researchers to analyze the results comprehensively. This study considers it impossible to obtain all empirical literature on the correlation between servitization and firm performance. Therefore, it is necessary to test this study’s publication bias to ensure the analysis results’ reliability. We adopted a funnel plot and fault-safety factor to verify whether there was a publication bias in empirical literature dealing with the servitization strategy-firm performance relationship. The results are shown in Figure 3.

Figure 3 shows that the effect sizes of most of the sample literature in this study are mainly concentrated at the top of the funnel plot. And their distributions are less discrete and more evenly distributed on both sides of the midline. This means that the sample literature obtained in this study has no significant publication bias and is representative. In addition, the fail-safe factor refers to how much literature with opposing or invalid results is needed to distort the original conclusion of the meta-analysis (Rosenthal, 1979). In other words, the *p*-value of the research result is greater than the confidence level, and the larger the value, the higher the reliability of the research conclusion (Liu et al., 2017). Our calculation results indicate that the fail-safe factor of this study is 85,833, which is much larger than the critical value of 550 (number of effect sizes*5+10). This further confirms that the conclusion obtained by meta-analysis in this study should be highly reliable.

### Heterogeneity

The heterogeneity test analyzes the degree of difference between multiple independent samples. If there is the heterogeneity, a random effects model needs to be selected to correct to ensure the consistency of the combined data. The heterogeneity test results are shown in Table 1. The *Q*-value reflects the heterogeneity degree of the sample literature effect values. The *I*^2^ represents the proportion of literature effect size difference ins the total difference. Experimental results show that the *Q*-value is 9481.51, much larger than the critical value of 107 *p* < 0.001. The value of *I*^2^ 98.871% was greater than the critical value of 50%, indicating that the difference was mainly due to the literature effect size. And only 1.129% of the observed variance is caused by sampling variance, which further indicates that there is heterogeneity in the sample. That is, there are potential moderator variables. A random effect model should be selected for subsequent data analysis.

**TABLE 1:** Heterogeneity test.

Model	n	K	r	95% CI		Heterogeneity			
				Upper limit	Lower limit	q	df	P	I^2^
Fixed effects	30278	108	0.317	0.325	0.309	9481.51	107	0.000	98.871
Fixed effects			0.365	0.434	0.292				

n = total sample size; K = number of sampled studies; r = effect size; 95% CI represents confidence interval at 5% significance level; *Q*, *df*, *P* and *I*^2^ are used to determine heterogeneity.

### Overall effect

Based on the heterogeneity test results, this study used a random effect model to test the overall effect, and the results are shown in Table 2. It can be seen that the comprehensive effect value between servitization and firm performance is 0.365, and the statistical result is significant (*p* < 0.001). This shows that servitization positively impacts firm performance, which supports Hypothesis H1. In addition, the combined effect values between product-oriented and customer-oriented servitization strategies and firm performance were 0.393 and 0.475, respectively, and the statistical results were all significant (*p* < 0.001). This shows that there is a significant positive correlation between product-oriented and customer-oriented servitization strategies and firm performance. Meanwhile, there is a stronger correlation between customer-oriented servitization strategies and firm performance. Hypothesis H2a and H2b are supported. These results suggest a moderately positive relationship between servitization and its different orientations and firm performance at the overall level. Although there are still some controversies in the existing research. From the perspective of a longer time span, a broader geographical scope, and larger sample size, the service-oriented strategy and its different orientations can effectively improve firm performance.

**TABLE 2:** Overall effect test.

Factor	n	K	r	95% CI		Z	P	Heterogeneity				Fail-safe *N*
				Upper limit	Lower limit			Q	df	p	I^2^	
Servitization strategy	30278	108	0.365	0.434	0.292	9.126	0.000	9481.510	107	0.000	98.971	85833
Product-oriented	5794	20	0.393	0.497	0.279	6.319	0.000	606.130	19	0.000	96.865	4459
Customer-oriented	6310	26	0.475	0.575	0.361	7.308	0.000	1222.211	25	0.000	97.955	1585

### Moderating effects

The overall effect results by Meta-analysis show that there was heterogeneity among the independent samples (*Q* = 9481.51). This means that the servitization–firm performance relationship is affected by potential moderator variables. According to the coding results, this study conducted meta binary analysis on the sample literature, and the results are shown in Table 3.

**TABLE 3:** Moderating effect test.

Factor	Sub-samples	n	K	r	95% CI		Z	P	Heterogeneity				Fail-safe *N*
					Upper limit	Lower limit			Q	df	P	I^2^	
Measurement factors	Financial performance	19317	50	0.278	0.398	0.149	4.142	0.000	7324.600	49	0.000	99.331	21475
	Non-financial performance	10961	58	0.436	0.503	0.364	10.697	0.000	1984.148	57	0.000	97.127	49222
Mediating variables	Exist	7633	35	0.416	0.496	0.329	8.574	0.000	1262.402	34	0.000	97.307	8165
	Inexistence	22645	73	0.340	0.434	0.238	6.269	0.000	8216.597	72	0.000	99.124	64588
Sampling area	Developed areas	19312	53	0.321	0.433	0.199	4.972	0.000	5584.577	52	0.000	99.069	30891
	Developing areas	10966	55	0.406	0.486	0.319	8.436	0.000	3545.292	54	0.000	98.477	37115
Market environment	Stable	1437	13	0.540	0.632	0.432	8.341	0.000	157.133	12	0.000	92.363	3023
	Volatile	28841	95	0.339	0.415	0.259	7.831	0.000	9131.416	94	0.000	98.971	98254
Technical level	High-tech	7678	33	0.450	0.599	0.270	4.579	0.000	4816.629	32	0.000	99.336	21478
	Traditional	22600	75	0.326	0.394	0.254	8.478	0.000	4236.986	74	0.000	98.253	49226
Firm scale	SMEs	23264	63	0.290	0.390	0.184	5.205	0.000	7400.958	62	0.000	99.162	36325
	Large firm	7014	45	0.463	0.546	0.371	8.800	0.000	1946.699	44	0.000	97.740	31620

From the results in Table 3, it can be seen that, from the perspective of performance measurement types, the comprehensive effect values of servitization strategy on firm financial performance and non-financial performance are 0.278 and 0.436, respectively. And the statistical results were all significant (*p* < 0.001). This shows that the servitization strategy has a more significant impact on the non-financial performance of firms, which supports Hypothesis H3.

In terms of the presence of mediating variables, the comprehensive effect values of the presence and absence of mediating variables were 0.416 and 0.340, respectively. And the statistical results were all significant (*p* < 0.001). This shows that the influence of servitization strategy on firm performance is more significant when there are mediating variables, which supports Hypothesis H4.

From the sampling areas, the comprehensive effect values of developed and developing areas were 0.321 and 0.406, respectively. And the statistical results were all significant (*p* < 0.001). This shows that the implementation of servitization strategies by manufacturing firms in developing areas significantly impacts firm performance, which supports Hypothesis H5.

In terms of market environment, the comprehensive effect values of the market stability period and market turbulence period were 0.540 and 0.339, respectively. And the statistical results were all significant (*p* < 0.001). This means that the effect of servitization strategy on firm performance is more significant in the period of market stability, and Hypothesis H6 is supported.

In terms of firm technical level, the comprehensive effect values of traditional manufacturing and high-tech manufacturing were 0.326 and 0.450, respectively. And the statistical results were all significant (*p* < 0.001). This shows that the implementation of servitization strategy in high-tech manufacturing firms has a more significant effect on the improvement of firm performance, which supports Hypothesis H7.

In terms of firm size, the comprehensive effect values for SMEs and large firms were 0.290 and 0.463, respectively. And the statistical results were all significant (*p* < 0.001). This indicates that the implementation of servitization strategy in large firms has a more significant effect on firm performance, which supports Hypothesis H8.

## Discussion

This study uses Meta-analysis to systematically analyze the relationship between servitization strategy and firm performance, given the role of multiple conditional variables. The results showed that servitization and its different orientation strategies exerted a positive impact on firm performance. Business models that integrate manufacturing and services are conducive to generating new value growth for firms, which improves business performance (Hu, 2016). As servitization develops, manufacturing firms’ original resources and capabilities can hardly support them, which may bring a short-term decline in business performance. However, the scale economies generated by services businesses that are increasingly diversified will continue to drive performance growth (Kastalli and Van Looy, 2013). Hypothesis H1 was supported. In addition, both product-oriented and customer-oriented servitization strategies positively impact firm performance, which supported hypotheses H2a and H2b. And the impact of customer-oriented servitization strategies on firm performance is more significant. In a more recent study by Visnjic et al. (2016), a customer-oriented servitization strategy has higher strategic value while potentially riskier for manufacturing firms.

Servitization strategy plays a more significant role in improving non-financial performance than financial performance. A recent study by Lexutt (2020) reiterates that financial metrics are not the only way to measure the success of servitization. Servitization success can also be achieved *via* non-financial performance, which directly impacts customer satisfaction and product value (Queiroz et al., 2020). Non-financial benefits from servitization strategies help manufacturing firms deal with increasing competitive pressures and changing customer needs. Besides, manufacturing firms often need to invest significant resources in the servitization process to develop new businesses, which may erode financial performance (Forkmann et al., 2017). The results supported hypothesis H3.

Servitization can have an indirect effect on firm performance through other variables. For example, the development of a servitization strategy needs more information technology support, which makes digitalization play a mediating role (Martín-Peña et al., 2019). Similarly, supply chain integration is also one of the mediating variables. Because servitization enhances the internal integration of firms with suppliers and customers, thus affecting firm performance (Shah et al., 2020). Such mediating variables, among many others, may be an effective way for manufacturing firms to achieve service-oriented transformation (Juhong et al., 2020). These results supported hypothesis H4.

Hypothesis H5 is supported, which suggests that manufacturing firm’s servitization contributes more significantly to performance in developing areas. Factors such as weak strength and late development are not only the disadvantages of manufacturing firms in developing areas, but also easily arouse their enthusiasm for servitization (Bao and Toivonen, 2015). Meanwhile, policy support from governments in developing areas can help manufacturing firms overcome external obstacles in their servitization transformation process (Vendrell-Herrero and Wilson, 2017).

Servitization strategy has a more significant effect on firm performance in a stable market environment. Because many uncertainties in turbulent marketing environments bring many risks to servitization transformation. Many firms may abandon servitization transformation to seek conservative business strategies to avoid risks. In addition, according to cost transaction theory, environmental uncertainty and information asymmetry can worsen the transaction difficulty of firms. Resulting in difficulties for firms to capture dynamic information related to customers in a timely manner and to accurately grasp customer demands (Zhang et al., 2019). These results were consistent with work by Ying et al. (2020), who pointed out that implementation of servitization strategies in highly uncertain markets by firms prone to ignore potential customer groups and miss innovation opportunities. Thus, hypothesis H6 is supported.

High-tech manufacturing firms implement servitization strategies significantly more effective in improving their performance. It confirms most scholars’ research conclusion (Coreynen et al., 2017). Products of high-tech manufacturing firms are often characterized by high innovation, high complexity, and high profitability. Which determines that most services businesses they provide will be more innovative, with more advanced methods and higher added value. Thus, the service business has a higher marginal substitution effect in this type of firm, which can bring higher performance to firms. Moreover, strong customer demand for high-tech services provides a huge market opportunity for its servitization transformation. Therefore, hypothesis H7 is supported.

Large manufacturing firms implement servitization strategies to improve their performance more significantly. It is consistent with the conclusion of Shin et al. (2022). From an internal perspective, large firms usually have various products, standardized management systems, and organized business processes. All these help firms cope with the problems that may arise in the servitization transformation process and achieve scale economies quickly. From an external perspective, large firms often have a wide market share, excellent customer relationships, and brand image. Which provides a foundation for its expansion into new service businesses. However, most SMEs do not have these advantages. Thus, it is difficult for them to realize the expected benefits through servitization strategy, which may be beyond their capacity (Mennens et al., 2018). These results support hypothesis H8.

Finally, based on the above analysis, this study found several reasons that may lead to inconsistent conclusion. Manufacturing firms need resources, capabilities, and corporate culture matching their servitization strategy to achieve their servitization development goals. Manufacturing firms must coordinate multiple internal and external factors before achieving servitization performance improvement. However, not all of these factors have been explored by scholars. Moreover, our study found that the presence of mediating variables may be one of the reasons for the discrepancy in the conclusion. Besides, the implementation of servitization strategy by large manufacturing firms seems to be more actionable, which also explains the divergent conclusion of the study from another perspective. It can be argued that the ignorance of moderating effect of firm size on the relationship between them contributes to the inconsistent conclusion of the study. Therefore, scholars should try to explore their relationship from multiple perspectives and conditions in follow-up research.

## Conclusion and limitation

This study answers the research questions we mentioned previously based on the results of a meta-analysis of 59 empirical articles on the relationship between servitization strategy and firm performance. It provides empirical evidence and reference for the research on the relationship between servitization strategy and their different orientations and firm performance. Where we first obtained 108 effect sizes and 30,278 independent samples by sampling and coding. Then, Meta-analysis was conducted to test the relationship between servitization strategy and their different orientations and firm performance. And the effects of measuring factors, mediating variables and sampling area, market environment, technical level, and firm scale on the relationship between servitization strategy and firm performance were also tested. The results support our hypotheses. Finally, this study discusses the data processing results. The main conclusion and implications are as follows:

Servitization transformation in manufacturing firms needs to be supported by efficiently operating management models. That facilitates its profitability by servitization, or it may cause negative effects. When manufacturing firms are equipped with resources and capabilities to deal with the challenges caused by servitization transformation, the customer-oriented strategy may be their best choice. Or they can start with basic services and develop to customer-oriented service strategy after accumulating enough capabilities. Additionally, manufacturing firms should comprehensively consider servitization strategies’ implications on financial and non-financial performance. Focusing on the long-term benefits and minimizing the impact of time lags in transforming non-financial performance into financial performance is important. Meanwhile, identifying opportunities and challenges in the market environment is important for manufacturing firms to seize timely service innovation opportunities. Traditional manufacturing firms require continuous improvement of technology innovation capabilities before they can utilize servitization to enhance their performance while safeguarding core competencies. Furthermore, manufacturing firms in developing areas can improve short-term benefits by applying the servitization concepts and technologies of manufacturing firms in developed areas. But they still need to consider their local context to explore the servitization transformation path.

Several limitations of this study need to be improved. First, to ensure the reliability of the study data, this study was strictly selected according to certain criteria to obtain high-quality literature. For example, the sample literature must be an empirical study related to servitization and firm performance, and the samples need to be independent of each other. This may result in some relevant empirical literature not being included in the analysis. Second, although this study comprehensively considered the moderating effects of multiple elements on the servitization strategy-firm performance relationship. But it remains difficult to identify all potential moderating variables. Therefore, there may be other influencing factors that remain unidentified, which could still lead to inconsistent conclusion. These limitations require improvement in subsequent studies.

## Data availability statement

The raw data supporting the conclusions of this article will be made available by the authors, without undue reservation.

## Author contributions

JW and KL: conceptualization. KL and LZ: methodology, software, and writing—review and editing. JW and LF: validation. LF: formal analysis, investigation, and supervision. LF and LZ: data curation. KL: writing—original draft preparation. JW: funding acquisition. All authors contributed to the article and approved the submitted version.
